# Hypothermic machine perfusion reduces the incidences of early allograft dysfunction and biliary complications and improves 1-year graft survival after human liver transplantation: A meta-analysis

**DOI:** 10.1097/MD.0000000000016033

**Published:** 2019-06-07

**Authors:** Yili Zhang, Yangmin Zhang, Mei Zhang, Zhenhua Ma, Shengli Wu

**Affiliations:** a Department of Medical Imaging, The First Affiliated Hospital of Xi’an Jiaotong University; b Department of Blood Transfusion, Xi’an Central Hospital; c Department of Hepatobiliary Surgery, The First Affiliated Hospital of Xi’an Jiaotong University, Xi’an, Shaanxi, P.R. China.

**Keywords:** hypothermic machine perfusion, liver, meta-analysis, static cold storage, transplant

## Abstract

**Background:**

The worldwide organ shortage continues to be the main limitation of liver transplantation. To bridge the gap between the demand and supply of liver grafts, it becomes necessary to use extended criteria donor livers for transplantation. Hypothermic machine perfusion (HMP) is designed to improve the quality of preserved organs before implantation. In clinical liver transplantation, HMP is still in its infancy.

**Methods:**

A systematic search of the PubMed, EMBASE, Springer, and Cochrane Library databases was performed to identify studies comparing the outcomes in patients with HMP versus static cold storage (SCS) of liver grafts. The parameters analyzed included the incidences of primary nonfunction (PNF), early allograft dysfunction (EAD), vascular complications, biliary complications, length of hospital stay, and 1-year graft survival.

**Results:**

A total of 6 studies qualified for the review, involving 144 and 178 liver grafts with HMP or SCS preservation, respectively. The incidences of EAD and biliary complications were significantly reduced with an odds ratio (OR) of 0.36 (95% confidence interval [CI] 0.17–0.77, *P* = .008) and 0.47 (95% CI 0.28–0.76, *P* = .003), respectively, and 1-year graft survival was significantly increased with an OR of 2.19 (95% CI 1.14–4.20, *P* = .02) in HMP preservation compared to SCS. However, there was no difference in the incidence of PNF (OR 0.30, 95% CI 0.06–1.47, *P* = .14), vascular complications (OR 0.69, 95% CI 0.29–1.66, *P* = .41), and the length of hospital stay (mean difference −0.30, 95% CI −4.10 to 3.50, *P* = .88) between HMP and SCS preservation.

**Conclusions:**

HMP was associated with a reduced incidence of EAD and biliary complications, as well as an increased 1-year graft survival, but it was not associated with the incidence of PNF, vascular complications, and the length of hospital stay.

## 1 Introduction

Liver transplantation is still one of the most effective treatments for end-stage liver diseases. However, the success of liver transplantation has been constrained by a persistent shortage of suitable donor organs. To reduce the gap between the need and availability of donors, extended criteria donor (ECD) livers, which exceed traditional limits for steatosis and/or donor age, or are procured via donation after cardiac death, are increasingly being used.^[1]^ However, these organs are most susceptible to the serious consequences of preservation-related injury, which include primary nonfunction (PNF), early allograft dysfunction (EAD), cholestasis, infectious complications, prolonged intensive care, and even a need for retransplantation.^[2]^ Many ECD livers are turned down because of the potential risk of preservation injury.^[3]^ Each year, the number of discarded livers is approximately 10% of all livers recovered worldwide.^[4]^

Static cold storage (SCS), currently the main technique used to preserve allograft liver in clinical practice, has been essentially unchanged during the last 3 decades. However, it is still not enough to prevent preservation related injury of the livers, especially for the ECD livers. Optimizing techniques for these liver grafts would help to increase the donor pool and under such a background, hypothermic machine perfusion (HMP) before liver transplantation has gained more and more attention in recent years. In this technique, a preservation solution containing metabolic substrates and other protective mediators are circulated through the donor organ, while flushing cytokines, proteins, and toxins out from the liver. This will attenuate the cytokine-mediated ischemia/reperfusion damage build-up that occurs within a statically preserved liver.^[5,6]^ Another advantage of HMP is that it enables the doctors to judge the acceptability of the graft by registering the pump parameters, such as flow and pressure, and analyzing the enzymes in the perfusate.^[7,8]^

In the field of kidney transplantation, the use of HMP has been correlated with improved graft and patient survival in a large, randomized trial^[9]^ and has been widely adopted.^[10,11]^ However, clinical trials of liver HMP are still in its infancy, although studies of HMP in animal models of liver transplantation have been promising.^[12–14]^ To better understand whether HMP could obtain better outcomes in human liver transplantation compared to SCS, we conducted a meta-analysis of the available studies. We assessed the impact of HMP on incidences of PNF, EAD, vascular complications, biliary complications, length of hospital stay, and 1-year graft survival. These data could help clinical transplant professionals to decide the best way to preserve human livers, especially ECD livers.

## 2 Materials and methods

### 2.1 Data sources and searches

**Figure 1:**
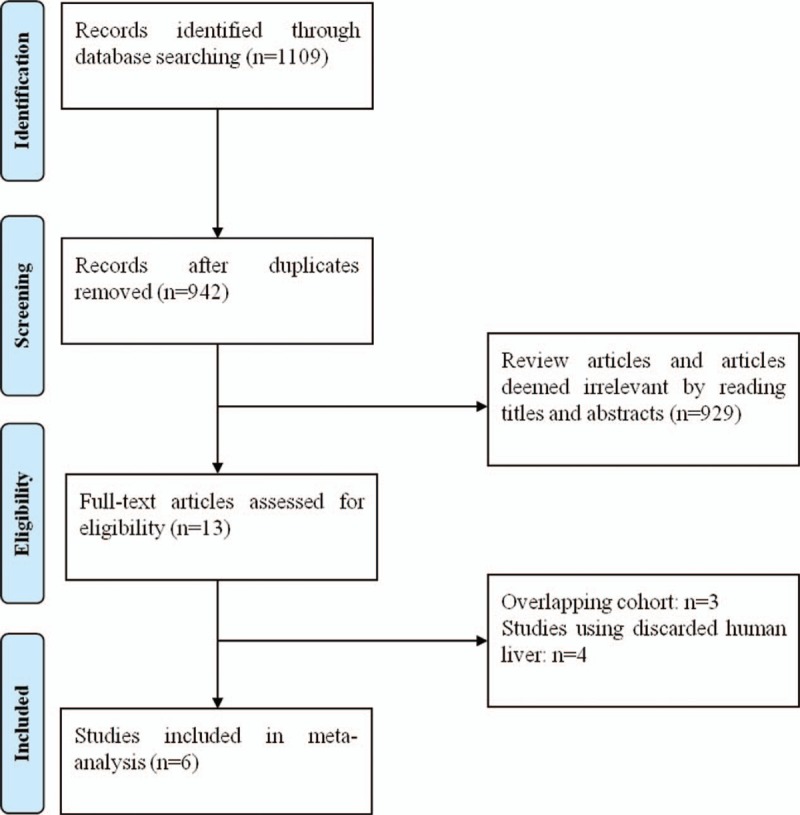
The preferred reporting items for meta-analyses protocol flowchart illustrating the selection of studies included in our meta-analysis.

A search of the PubMed, ISI Web of science, Springer, and Cochrane Library databases was performed with the following search terms as free-text terms as well as MeSH terms: machine perfusion, hypothermic machine perfusion, cold storage, static cold storage, liver, and hepat∗. The process of identifying papers for inclusion is shown in Figure 1. The search was conducted in January 2019. A manual search of the references of the relevant publications was also performed. No language restrictions were imposed.

This study was reviewed and approved by the Ethics Committee, Xi’an Jiaotong University Health Science Center.

### 2.2 Selection criteria

Studies reporting outcomes of human liver transplantation using HMP preservation versus SCS were included in this meta-analysis. Exclusion criteria were:

(1) overlapping studies from the same institution (avoid duplication);
(2) studies that used discarded human livers;
(3) studies that included livers from simultaneous liver-kidney transplants;
(4) HMP or SC solutions with additional drugs, for example, α-tocopherol;
(5) animal studies; and
(6) review articles.

### 2.3 Data extraction

The data extracted include: the first author, publication year, study design, study period, sample size, preservation solution, donor and recipient age, lab model for end-stage liver disease score, warm ischemic time (WIT), total cold ischemic time (CIT), machine perfusion time, cannulation site, flow rate, active oxygenation, PNF, EAD, vascular complications, biliary complications, hospital length of stay, and 1 year graft survival rate.

### 2.4 Quality assessment

Two investigators independently read the titles and abstracts of potential studies, and then the full texts of eligible studies. The Newcastle–Ottawa scale (NOS)^[15]^ was used to evaluate the quality of each observational study. According to the NOS score standard, each study is judged on 3 broad perspectives: selection, comparability, and exposure for case–control studies. Studies with total scores ≥7 were defined as high quality, 5 to 6 as moderate quality, and ≤4 as low quality.

### 2.5 Data synthesis and analysis

The meta-analysis was performed using the statistical software RevMan5.1 (The Cochrane Collaboration). Pooled odds ratios (ORs) with 95% confidence interval (CI) were used as the effect indicator for the dichotomous variables, and pooled weighted mean differences (MDs) were used for the measurement data. A *P* value < .05 was considered a significant difference between the 2 groups. Heterogeneity in all of the included studies was evaluated by *X*^2^ and *I*^2^ statistical tests. A random effect model was adopted when *P* < .05 or *I*^2^ > 50%, and a fixed-effect model was used when *P* > .05 or *I*^2^ < 50%. Sensitivity analysis was used to assess the stability of results by systematically removing each study and reassessing the significance.

## 3 Results

### 3.1 Search results and included studies

Overall, 1109 potentially relevant articles were retrieved according to the search strategy. Among these, 929 were excluded after reading the title and abstract and 167 were excluded due to duplication. Thus, 13 studies were potentially eligible for this systematic review. However, 7 studies were further excluded due to overlapping cohort (n = 3) and the use of discarded human liver (n = 3). Finally, 6 studies comparing HMP to SCS were included in this review, involving 144 and 178 human liver grafts with HMP and SCS preservation, respectively^[16–21]^ (Fig. 1). Although 4 of the studies are from 2 institutions (Ref 16 and 18, Ref 17 and 19), they are not duplicates.

All 6 studies were cohort study and 3 of them were prospective study, while the remaining 3 were retrospective study. The years of publication spanned from 2010 to 2019. The included studies were conducted in 4 countries: 2 in the United States, 2 in Switzerland, 1 in Netherland, and 1 in the United Kingdom and Switzerland. None of the 6 studies was considered low quality and the study characteristics are presented in Table 1.

**Table 1:**
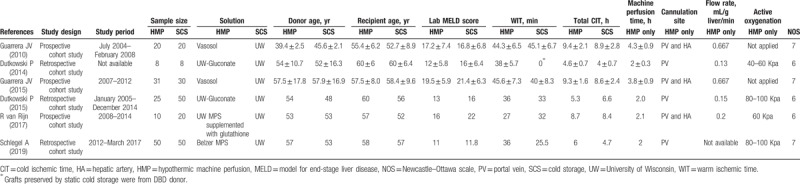
Characteristics of the included studies.

### 3.2 Outcomes

**Figure 2:**
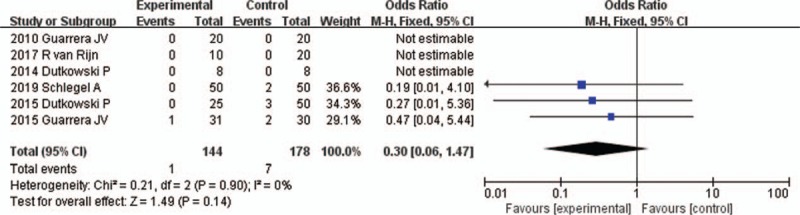
PNF rates for liver allografts preserved by HMP versus SCS in all studies. CI = confidence interval, HMP = hypothermic machine perfusion, M-H = Mantel–Haenszel test, PNF = primary nonfunction, SCS = static cold storage.

All 6 studies reported the incidence of PNF^[16–21]^ (Table 2). PNF was determined as retransplantation or death within 7 days of transplantation in one of the studies.^[20]^ The other 5 studies did not provide a definition of PNF. The fixed-effects model was adopted, as the heterogeneity analysis had not shown a significant difference. The meta-analysis showed that the incidence of PNF was not significantly different between HMP and SCS preservations (OR 0.30, 95% CI 0.06–1.47, *P* = .14) (Fig. 2).

**Table 2:**
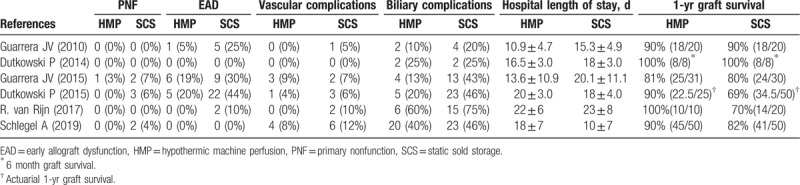
Patient outcomes of the included studies.

**Figure 3:**
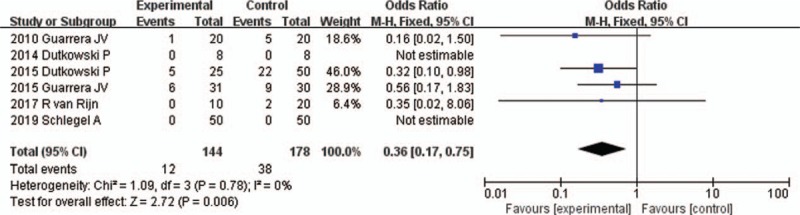
EAD rates for liver allografts preserved by HMP versus SCS in all studies. CI = confidence interval, EAD = early allograft dysfunction, HMP = hypothermic machine perfusion, M-H = Mantel–Haenszel test, SCS = static cold storage.

All 6 studies reported the incidence of EAD^[16–21]^ (Table 2). Four of the studies defined EAD as the presence of at least one of the following at 7 days after liver transplantation: serum bilirubin ≥10 mg/dL, or international normalized ratio ≥1.6, or alanine aminotranferease >2000 in the first 7 postoperative days.^[16,18–20]^ The other 2 studies did not provide a definition of EAD. The fixed-effects model was adopted because the heterogeneity analysis did not show a significant difference. We found that the incidence of EAD was significantly reduced in the HMP preservation compared with SCS preservation with an OR of 0.36 (95% CI 0.17–0.75, *P* = .006) (Fig. 3).

**Figure 4:**
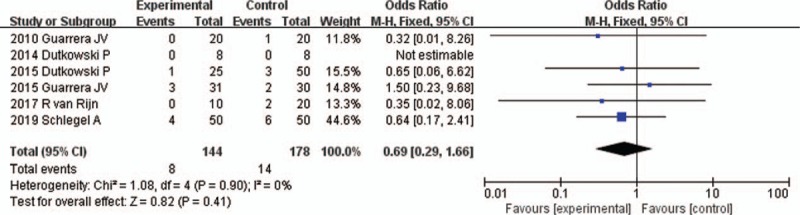
Vascular complication rates for liver allografts preserved by HMP versus SCS in all studies. CI = confidence interval, HMP = hypothermic machine perfusion, M-H = Mantel–Haenszel test, SCS = static cold storage.

All 6 studies reported the incidence of vascular complications^[16–21]^ (Table 2). None of the study provided a definition of vascular complications. In Ref 16, the vascular complications referred to hepatic artery stenosis. In Ref 18, the vascular complications referred to portal vein thrombosis and hepatic artery thrombosis. In Refs 19 and 20, the vascular complications referred to hepatic artery thrombosis. In Ref 21, the vascular complications referred to arterial complications. No vascular complications occurred in Ref 17. The fixed-effects model was adopted, as the heterogeneity analysis had not shown a significant difference. The meta-analysis showed that the incidence of vascular complications was not significantly different between HMP and SCS preservations (OR 0.69, 95% CI 0.29–1.66, *P* = .41) (Fig. 4).

**Figure 5:**
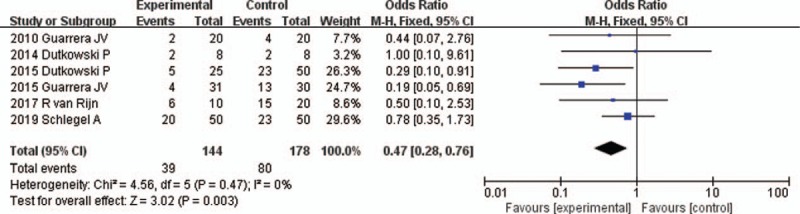
Biliary complication rates for liver allografts preserved by HMP versus SCS in all studies. CI = confidence interval, HMP = hypothermic machine perfusion, M-H = Mantel–Haenszel test, SCS = static cold storage.

All 6 studies reported the incidence of biliary complications^[16–21]^ (Table 2). None of the study provided a definition of biliary complications. In Refs 16, 17, and 18, the biliary complications referred to bile leakage and biliary stricture. In Ref 19, the biliary complications referred to bile leakage, biliary stricture, and ischemic cholangiopathy. In Refs 20 and 21, the biliary complications referred to bile leakage, biliary stricture, and biliary cast formation. The fixed-effects model was adopted, as the heterogeneity analysis had not shown a significant difference. The meta-analysis showed that the incidence of biliary complications was significantly reduced in the HMP preservation compared with SCS preservation (OR 0.47, 95% CI 0.28–0.76, *P* = .003) (Fig. 5).

**Figure 6:**
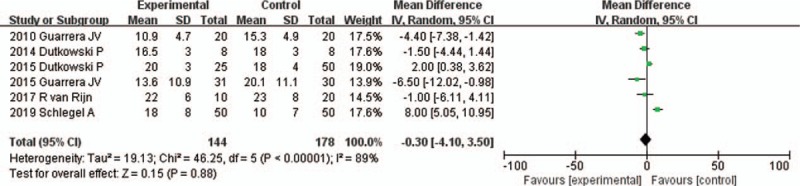
The length of hospital stay for liver allografts preserved by HMP versus SCS in all studies. CI = confidence interval, HMP = hypothermic machine perfusion, M-H = Mantel–Haenszel test, SCS = static cold storage.

All 6 studies reported the length of hospital stay^[16–21]^ (Table 2). The random-effects model was adopted, as the heterogeneity analysis had shown a significant difference. The meta-analysis showed that the length of hospital stay was not significantly different between HMP and SCS preservation (MD −0.30, 95% CI −4.10 to 3.50, *P* = .88) (Fig. 6).

**Figure 7:**
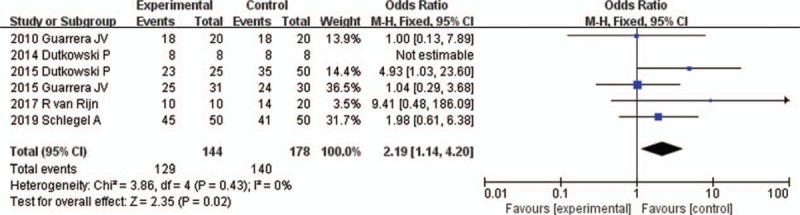
One-year graft survival rates for liver allografts preserved by HMP versus SCS in all studies. CI = confidence interval, HMP = hypothermic machine perfusion, M-H = Mantel–Haenszel test, SCS = static cold storage.

All 6 studies reported the incidence 1-year graft survival^[16–21]^ (Table 2). The fixed-effects model was adopted, as the heterogeneity analysis had not shown a significant difference. The meta-analysis showed that the incidence 1-year graft survival was significantly increased in HMP preservation compared to SCS preservation (95% CI 1.14–4.20, *P* = .02) (Fig. 7).

### 3.3 Sensitivity analysis

Sensitivity analysis was performed by sequentially deleting each individual data set for each factor analyzed to evaluate the stability of the results.^[22]^ The results showed that no individual study significantly influence the pooled ORs or MDs, except for the study of Dutkowski et al^[19]^ for EAD, as well as the studies of Dutkowski et al^[19]^ and van Rijn et al^[20]^ for 1-year graft survival. The sensitivity analysis that excluded these data sets resulted in significant changes to the incidence of EAD and 1-year graft survival between HMP preservation and SCS preservation.

### 3.4 Publication bias

Given the limited number of studies (n = 6) in the current meta-analysis, we took no formal steps to determine publication bias, since any formal method would have had little power.

## 4 Discussion

Although HMP remains investigational in clinical liver transplantation, pilot studies had demonstrated its feasibility, safety, and efficacy with diminished peak injury markers, lower length of hospital stay, a trend toward less EAD, and fewer biliary complications.^[5,7,16]^ However, due to the limited number of cases in individual studies, the differences in most preservation parameters did not reach a significant level and more results are required with adequate power to confirm these observations. As far as we know, there were only 6 clinical liver transplantations comparing HMP with SCS till now and no meta-analyses comparing the 2 preservation methods were reported previously.

EAD is defined as a serious complication after liver transplantation and the incidence of EAD is strongly correlated with graft loss or mortality of patients.^[23–25]^ A recent study has reported the risk of liver graft failure at 90 days post transplantation was 5.2 times higher in recipients with EAD than those without EAD, and the risk factors associated with EAD include graft type and size, preoperative bilirubin, portal reperfusion pressure, donor age, and donor body mass index.^[26]^ In this meta-analysis, we found that HMP significantly reduced the incidence of EAD, although the sensitivity analysis could not determine a significant difference when the data set in the study of Dutkowski et al^[19]^ was removed. Two studies in this meta-analysis showed that no EAD occurred,^[17,21]^ while the other 4 showed a tendency of decreased EAD in the HMP group.^[16,18–20,22]^ Especially, the study of Dutkowski et al demonstrated a strong tendency of decreased EAD in the HMP group (*P* = .05). That might be the explanation for the lack of significant difference in pooled incidence of EAD once the data set in Ref 19 was deleted.

Up to 30% of patients develop biliary complications after liver transplantation that range from strictures to leaks^[27]^ and may become a significant cause of mortality, ranging from 6% to 12.5%.^[28,29]^ In this meta-analysis, the incidence of biliary complications was identified to be significantly reduced in HMP group when compared with SCS group. In addition, the sensitivity analysis showed the same effect by sequentially deleting each individual data set. Among them, 1 study showed the incidence of biliary complications was same in the 2 groups,^[17]^ 2 studies showed a significantly less biliary complications in the HMP group versus the SCS group,^[18,19]^ while the other 3 showed a tendency of less biliary complications in the HMP group.^[16,20,21]^ The pooled results of the decreased incidences of EAD and biliary complications may be attributed to the better flushing and continuous circulation of adequate oxygen, adenosine triphosphate substrates, and vasodilators to the peribiliary vascular microcirculation during HMP.^[30–32]^

Another significant finding is that the 1-year graft survival was increased obviously in HMP preservation compared to SCS, although the sensitivity analysis could not determine a significant difference when the data set in the study of Dutkowski et al^[19]^ or van Rijn et al^[20]^ was removed. Three studies in this meta-analysis showed the 1-year graft survival was same or similar in the 2 groups,^[16–18]^ while the other 3 showed a tendency of increased 1-year graft survival in the HMP group.^[19–21]^ Especially, the studies of Dutkowski et al and van Rijn et al demonstrated a strong tendency of increased 1-year graft survival in the HMP group (*P* = .05 and *P* = .14, respectively). That might be the explanation for the lack of significant difference in pooled incidence of EAD once the data set in Ref 19 or Ref 20 was deleted. HMP significantly reduced proinflammatory cytokine expression and relieved the downstream activation of adhesion molecules and migration of leukocytes, including neutrophils and macrophages in human liver transplantation.^[6,7]^ All these mechanisms promote an early improvement in liver function, which might allow easier titration of calcineurin inhibitors to therapeutic levels, in turn facilitating a significant increase in 1-year graft survival.^[16,18]^

Besides, we found that HMP preservation was not associated with the reduction in the incidences of PNF and vascular complications, as well as length of hospital stay.

There are some potential limitations to this meta-analysis, which may increase the possibility of publication bias and affect the final result. First, this meta-analysis contained only 6 studies and the number of cases was still limited for this special subject. Second, all of them were cohort studies. Although they provided the best evidence available on this subject, the nonrandomized studies might have resulted in an unbalanced selection of patients. Third, there was heterogeneity in the graft quality or donor status, including the length of WIT and CCT, donor age, steatosis, types of machine perfusion solution, perfusion route, perfusion pressure, and with or without active oxygenation, which was correlated with the study design and the preferences of individual hospitals.

In conclusion, this meta-analysis demonstrates that HMP is associated with a reduced incidence of EAD and biliary complications, as well as an increased 1-year graft survival compared to SCS for liver transplantations, but it was not associated with the incidence of PNF, vascular complications, and the length of hospital stay. However, due to the limitations of this analysis, further large multicenter randomized controlled trials are needed to confirm this conclusion.

## Author contributions

**Conceptualization:** Shengli Wu.

**Data curation:** Yili Zhang, Yangmin Zhang.

**Formal analysis:** Mei Zhang.

**Funding acquisition:** Shengli Wu.

**Methodology:** Mei Zhang, Zhenhua Ma.

**Writing – original draft:** Yili Zhang.

**Writing – review and editing:** Shengli Wu.
